# Using functional genomics to decipher the complexity of microbial pathogenicity

**DOI:** 10.1007/s00294-016-0576-4

**Published:** 2016-02-22

**Authors:** Maisem Laabei, Ruth Massey

**Affiliations:** Department of Biology and Biochemistry, University of Bath, Bath, BA2 7AY UK

**Keywords:** *Staphylococcus aureus*, Toxicity, GWAS, Evolutionary trade-offs, Bacteraemia

## Abstract

From the first identification of bacteria as a causative agent of disease, researchers have been developing methods and techniques to understand their pathogenic processes. For decades, this work has been limited to looking at a small number of genetically manipulatable isolates in in vitro assays or animal models of infection. Despite these limitations such work has facilitated the development of successful therapeutic strategies, most notably vaccines that target specific virulence-related features. There are however many antimicrobial resistant pathogens for which vaccination strategies have not worked, as we simply do not know enough about how they cause disease. We are however at the dawn of a new era in the study of microbial pathogenicity, where large collections of bacteria isolated directly from human infections can be sequenced and assayed to identify the bacterial features that affect disease severity in humans. Here, we describe our attempt to perform such a study focussed on the major human pathogen *Staphylococcus aureus*, which demonstrates the step changes such approaches can make to understanding microbial pathogenicity.

*Staphylococcus aureus* is the primary cause of a wide range of human infections, including septicaemia, abscesses and endocarditis (Lowy 1998). Although the rates of methicillin-resistant *S. aureus* (MRSA) are declining in certain European hospitals, a recent study showed that the incidence of MRSA bacteraemia was greater than 25 % in over one-third of European countries (Johnson 2011). In the United States, the rate of MRSA infection has decreased since 2006 (Klein et al. 2007); however, the proportion of those infections caused by the highly virulent, sequence type 8, USA300 clone has risen sharply (Jarvis et al. 2012; Rhee et al. 2015). USA300 is the predominant clone causing community-acquired infections in otherwise healthy individuals, and is now considered endemic in many US communities and hospitals (David and Daum 2010; Rhee et al. 2015). Even with increased surveillance, improved patient management and treatment regimes, *S. aureus* remains a major public health concern (Johnson 2011; Jarvis et al. 2012). As a response to the severe threat *S. aureus* possesses to global human health, a deeper understanding of its pathogenesis at the genetic and molecular level is warranted.

*Staphylococcus aureus* is both a major human pathogen and resident of the normal microflora. For decades, it has been considered an opportunistic pathogen, where breaches in host immunity are considered the route through which it transitions from being a coloniser to an invasive pathogen. This suggests that disease onset and severity are wholly driven by the host, where given identical opportunity two genetically distinct *S. aureus* isolates will cause identical disease. However, the fact that we have globally successful clones of *S. aureus* circulating and hyper-virulent strains causing a significant burden of disease suggests that specific bacterial features contribute significantly to the onset and severity of disease. Until we understand the effect of these on disease in humans we will not have a full picture of this microbe’s pathogenicity.

*Staphylococcus aureus* possesses an extensive repertoire of virulence factors facilitating its dual life-style as a commensal and a pathogen (Foster and Hook 1998; Vandenesch et al. 2012; Vorkapic et al. 2015), where the secretion of toxins is classically considered an essential element in the development of infection (Dinges et al. 2000). The staphylococcal scientific literature is abundant with many excellent studies involving the mechanism of action, host receptor tropism and regulation of the many toxins secreted by *S. aureus* (Vandenesch et al. 2012; Ibarra et al. 2013; DuMont and Torres 2014). At present, we are at a stage where it is affordable to rapidly and accurately sequence large numbers of clinical isolates, resulting in new opportunities to study pathogen evolution, epidemiology and antibiotic resistance (Didelot et al. 2012). The question that has been presented is whether this revolution in next-generation sequencing and abundance in genomic information can aid in our understanding of the fundamental basis of *S. aureus* virulence.

As the secretion of toxins is an integral part of the *S. aureus* virulence repertoire, we focussed on investigating the molecular and genetic basis of toxin secretion. Here, we combined whole-genome sequencing and phenotypic data, developing a genome-wide association study (GWAS) to detail how polymorphisms affect bacterial cytotoxicity. In two recent publications, we document the functional genomics approach we undertook to determine which mutations affected this phenotype. In our first paper ‘Predicting the virulence of MRSA from its genome sequence’ (Laabei et al. 2014), we explored the use of GWAS to identify genes, acting singularly or in epistasis, which we predicted to have an impact of toxicity, using the globally important, widely-disseminated ST239 lineage (Laabei et al. 2014). From our list of 121 statistically significant polymorphisms affecting toxicity, we sought to functional verify 13 of these loci, resulting in the description of four previously uncharacterised regions of the ST239 genome, that when disrupted significantly impact on toxicity. Additionally, by adopting a machine learning approach using a subset of these polymorphisms (unique SNPs and one SNP from each group of SNPs in linkage disequilibrium) we were able to make predictions on the toxicity of these strains using sequencing data with high accuracy (>85 %) (Laabei et al. 2014).

*Staphylococcus aureus* gene expression is tightly controlled and has evolved to be energetically efficient. The secretion of toxins, generally considered to be positively associated with increased disease severity, comes at a high energetic price and is readily switched off in vitro (Somerville et al. 2002) with recent observational studies suggesting that this also occurs in vivo (Fowler et al. 2004; Rose et al. 2015). In our most recent paper, ‘Evolutionary trade-offs underlie the multifaceted virulence of *S. aureus*’ (Laabei et al. 2015), we adopted our functional genomics approach to understand and define mutations associated with this reduction in toxicity. Therein, we describe the elucidation of six novel loci that when mutated significantly decrease toxicity, highlighting the complex nature of *S. aureus* toxin regulation (Laabei et al. 2015). The decrease in toxicity was significantly associated with strains originating from bacteraemia, compared to strains from skin and soft tissue infections (SSTIs) or carriage strains. Previous groups showed that clinical strains isolated from the blood had increased defective mutations in the accessory gene regulator (Agr) operon, the main quorum sensing system which regulates toxins in *S. aureus* and had increased resistance to vancomycin (Sakoulas et al. 2005). Interestingly, in this collection of isolates there was no difference in the number of Agr mutations present in the carriage/SSTI group compared to the bacteraemia group, and no significant difference between vancomycin resistance between the two groups (data not published).

We further investigated several hypotheses as to why a decrease in toxicity may be selected for and associated with bacteraemia. By measuring growth dynamics and relative fitness, we found that low-toxic isolates were significantly more fit when grown in the presence of serum than high-toxic isolates. Given the extreme bottleneck bacteria have to traverse to establish a bloodstream infection, our hypothesis is that a reduction in toxicity by mutation of various genes increases its relative fitness compared to high-toxic strains due to the energy cost of toxin production.

The discovery and elucidation of novel virulence regulators has been an immensely important feature of understanding *S. aureus* pathogenesis. To the best of our knowledge there are approximately 30 known virulence regulators, including two-component systems, DNA-binding proteins and regulatory RNAs (Priest et al. 2012; Tuchscherr and Loffler 2015). Virulence factors’ expression in *S. aureus* occurs in a temporal growth-phase-dependent manner employing multiple arms of this complex regulon at any one time. In the past 2 years, as a result of our functional genomics approach a further 10 genetic loci have been shown to be affected by *S. aureus* virulence, specifically toxicity. From our unpublished work, we have identified a further three loci important in the regulation of toxicity, of which we are currently characterising. Following with this theme, and focusing on *S. aureus* biofilm activity, we have identified two novel regions that increase biofilm formation following gene inactivation. These preliminary forays into applying functional genomics approaches to microbial pathogens clearly illustrate how fruitful this can be.

The functional genomics approach we have developed makes use of assaying large numbers of clonally related clinical strains. This methodology has not only allowed us to make significant leaps in understanding the vast regulon that affects toxicity, and virulence in general, but has highlighted that intra-clonally, strains have highly variable toxicity levels, a feature that has not been shown to this extent before. The functional genomics approach allows us to better understand pathogen biology, shown here by the discovery of the trade-off which exists between *S. aureus* toxicity and fitness in serum which we hypothesise is an essential step in the progression of bacteraemia. As we are entering the next-generation sequencing era, whereby the cost of sequencing a single strain has reduced dramatically, whilst the accuracy has increased significantly, we desperately need a strategy to understand this abundance in genomic information and how it impacts on pathogen behaviour. By combining large-scale sequencing with comprehensive phenotypic studies, we can begin to map the entire virulence pathway, identifying virulence signatures responsible for hyper-virulence. These strategies may play a central role in developing the holy-grail of patient care, personalised medicine, whereby detailed patient data and bacterial genome sequences will be used in combination to optimise treatment and improve the outcome for patients.
